# Role of patient sex in prescribing long-term beta blockers after myocardial infarction in German-speaking European primary care settings: a cross-sectional online survey using case vignettes

**DOI:** 10.1136/bmjopen-2026-125086

**Published:** 2026-10-23

**Authors:** Rahel Grindat, Katharina T Jungo, Kali Tal, Christine Brütting, Patrick Erber, Mahmoud Moussa, Erika Zelko, Sven Streit, Martina Zangger

**Affiliations:** 1 Institute of Primary Health Care (BIHAM), University of Bern, Bern, Switzerland; 2 Center for Healthcare Delivery Sciences and Division of Pharmacoepidemiology and Pharmacoeconomics, Department of Medicine, Brigham and Women’s Hospital and Harvard Medical School, Boston, Massachusetts, USA; 3 Institute of General Practice, Faculty of Medicine, Martin Luther University of Halle Wittenberg, Halle (Saale), Saxony-Anhalt, Germany; 4 Division General and Family Medicine, Department of General Health Studies, Karl Landsteiner University of Health Sciences, Krems, Austria; 5 Department of Primary Care Medicine, Medical University of Vienna, Vienna, Austria; 6 Institute for General Medicine, Johannes Kepler University Linz, Linz, Austria; 7 Graduate School for Health Sciences, University of Bern, Bern, Switzerland

**Keywords:** Myocardial infarction, Deprescriptions, Primary Care

## Abstract

**Abstract:**

**Objectives:**

To examine whether general practitioners’ (GPs’) reported willingness to deprescribe long-term beta blockers after myocardial infarction with preserved left ventricular function differs by patient sex and to identify factors that influence the decision to deprescribe.

**Design:**

Cross-sectional online survey using case vignettes differing by patient sex, time since myocardial infarction (3 months, 5 years, 10 years) and presence of side effects.

**Setting and participants:**

GPs in primary care in Switzerland, Germany and Austria across five study sites.

**Primary and secondary outcome measures:**

The primary outcome was the proportion of GPs choosing to deprescribe beta blockers in different hypothetical case vignettes. Secondary outcomes included factors associated with expressing willingness to deprescribe in multivariable regression models and reasons for deprescribing.

**Results:**

185 GPs (35.7% female (n=66); mean age 50.7 years (SD 10)) completed the questionnaire (average 61.7 per country (59–66 per country)). In the vignettes without side effects, the proportion of patients for whom GPs reported to be willing to deprescribe increased with time since myocardial infarction (3 months: 23% for male patients and 31% for female patients; 5 years: 46.9% for male and 46.0% for female patients; 10 years: 74.5% for male and 73.6% for female patients). In the presence of side effects, willingness to deprescribe increased (3 months: 63% for male and 79% for female patients; 5 years: 75% for male and 84% for female patients; 10 years: 87% for male and 89% for female patients). Time since myocardial infarction (5 years since myocardial infarction: risk ratio (RR) 1.3, 95% CI 1.2 to 1.5; 10 years: RR 1.7, 95% CI 1.6 to 1.9, reference: 3 months), presence of side effects (RR 1.6, 95% CI 1.5 to 1.8; reference: no side effects) and country of practice were associated with deprescribing (Austria: RR 1.1, 95% CI 1.0 to 1.3; Switzerland: RR 1.2, 95% CI 1.1 to 1.4, reference: Germany). Male patient sex was associated with a lower likelihood of deprescribing compared with female sex (RR 0.9, 95% CI 0.8 to 1.0). Frequently cited reasons for deprescribing included quality of life and perceived balance of benefits and harms, with similar reasons reported across male and female patient vignettes.

**Conclusion:**

Patient sex showed a small association with GPs’ willingness to deprescribe long-term beta blockers, whereas willingness to deprescribe varied substantially across clinical scenarios, particularly with longer time since myocardial infarction and the presence of side effects.

**STRENGTHS AND LIMITATIONS OF THIS STUDY**

- This cross-sectional survey used standardised case vignettes, enabling direct comparison of deprescribing decisions across predefined hypothetical clinical scenarios.
- Inclusion of general practitioners (GPs) practising in primary care settings of three European countries allowed exploration of practice variation across healthcare systems but reduces generalisability beyond primary care settings in German-speaking countries.
- Decisions were based on hypothetical case vignettes, which may not reflect real-world clinical behaviour.
- Convenience sampling through professional networks may have introduced selection bias, potentially favouring GPs with a particular interest in deprescribing.
- The small sample size limits statistical power.

## Introduction

 Beta blockers are among the most frequently prescribed cardiovascular drugs and are often prescribed for life.^1^ Beta blockers have been used to treat myocardial infarction (MI) since the late 1980s and randomised controlled trials affirmed that they reduce mortality and morbidity.^2^ These trials were conducted before the introduction of reperfusion and secondary prevention drugs, including antiplatelets and statins, which were found to reduce heart failure.

The availability of newer therapies raises questions about the continued benefit and optimal duration of beta blockers for cardiovascular patients whose left ventricular ejection fraction (LVEF) has been preserved and whose other conditions do not indicate beta blocker therapy.^3–6^ More recent randomised controlled trials have questioned whether beta blocker therapy had a continuing benefit beyond the first year in patients who survived MI,^7 8^ and there is some debate about whether their use should be continued.^9^

Physicians must balance evidence-based recommendations against the realities of ageing populations and treatment burdens^10^ and general practitioners (GPs) are encouraged to reduce or halt medications when potential harms outweigh expected benefits. But European and American guidelines give inconsistent advice about the ideal length of beta blocker therapy, creating uncertainty for clinicians who must help patients make long-term management decisions^11–13^ and help them understand when and how to discontinue long-term therapies.^14^ The 2023 European Society of Cardiology guidelines recommend considering routine beta blocker therapy after acute coronary syndrome regardless of LVEF, whereas the 2025 American College of Cardiology/American Heart Association guideline states that the optimal duration of beta blocker therapy remains unclear in patients with preserved LVEF.^15 16^

To optimise treatment and reduce medication-related harm, the concept of deprescribing has gained increasing attention in recent years. Deprescribing has been defined as the process of withdrawing medications that are inappropriate or no longer provide clear benefit, with the goal of managing polypharmacy and improving patient outcomes.^17^ In the context of long-term beta blocker therapy after MI, deprescribing may be considered when the expected benefits of treatment become uncertain or are outweighed by treatment burden and potential harms.

Several factors may influence whether GPs decide to deprescribe long-term beta blocker therapy. Side effects may play an important role in this decision. Common side effects of beta blockers include fatigue, bradycardia and sexual dysfunction.^9 18 19^ Though we know of no studies of sex-specific adverse profiles, men suffer sexual dysfunction more often than women.^20^ Women on beta blockers have higher plasma concentrations than men and, if their dosage is not adjusted, may experience adverse drug reactions more frequently.^21^ In addition to biological differences, GPs may also perceive and interpret side effects differently, depending on whether their patients are men or women.

Sex and gender bias in cardiovascular diseases remains a pervasive issue, affecting diagnosis, treatment and outcomes.^22^ For example, women patients with coronary artery disease are less likely to receive lipid-lowering medication.^23^ Likewise, women with acute coronary syndrome and obstructive coronary artery disease are less likely to receive aspirin, beta blockers and statins at discharge^24^ even though they have higher risk profiles and mortality rates. Various studies demonstrate that medical personnel are less likely to treat women according to evidence-based guidelines, possibly because women are underrepresented in clinical trials.^23 25^ Other studies have shown that unconscious gender bias can subtly influence clinical reasoning and decision-making.^22 26^

We know little about how such biases affect GPs’ decisions to deprescribe beta blockers for cardiovascular patients. The publication reporting the ‘Long-term beta blocker prescribing after myocardial infarction in European primary care (PRACTITIONER)’ study investigated factors associated with GPs’ willingness to deprescribe long-term beta blockers after MI but did not examine the role of patient sex.^27^ A comparison according to patient sex was possible only in Austria, Germany and Switzerland, where otherwise identical male and female versions of the case vignettes were administered. In these three countries, participants were randomised to receive either version. The male-patient vignette was developed specifically for this substudy and yielded additional data not included in the main PRACTITIONER analysis. In this study, we therefore investigated whether patient sex was associated with GPs’ willingness to deprescribe beta blockers in this clinical context.

## Aims

Our primary aims were to determine how GPs report to manage beta blockers for patients who had had an MI and whether their patient’s sex influenced their decision to deprescribe and to identify other factors that influenced the GPs’ decisions to prescribe or deprescribe beta blockers.

## Methods

### Design

This was an exploratory, cross-sectional substudy of the PRACTITIONER study.^27^ PRACTITIONER investigated the prescribing and deprescribing decisions of GPs across 20 countries in Europe by presenting them with case vignettes as part of an online survey. In this substudy, we analysed data from GPs in Austria, Germany and Switzerland, where additional data were collected using male-patient versions of the case vignettes specifically developed to enable a comparison according to patient sex.

For feasibility reasons we were only able to do this additional data collection in German-speaking countries. Surveys presented standardised clinical vignettes describing patients after MI who had normal left ventricular function. The goal of the survey was to determine whether GPs would continue or discontinue long-term beta blocker therapy under varying conditions (eg, presence/absence of side effects, time since MI).

### Population and setting

Participants were practicing GPs from Austria, Germany and Switzerland. Active professional status was the only inclusion criterion and there were no exclusion criteria. We recruited national coordinators through the European General Practice Research Network and the study team’s personal contacts, who then invited GPs to participate via existing networks (ie, professional associations, academic primary care networks, mailing lists, local GP groups). In each country there could be more than one national coordinator, who used a convenience and snowballing sampling strategy to recruit potential participants. Coordinators received instructions for distributing the survey link within their networks. The wording and mode of the invitations varied between coordinators and recipients and were not standardised. Because the survey link was distributed openly through professional networks without a fixed sampling frame, the numbers of individuals who received the invitation and of unique visitors to the first survey page were unavailable. Consequently, view and participation rates could not be calculated. Participation was voluntary, and no incentives were offered. Anyone who received the link and reported to fulfil the eligibility criterion about practicing in primary care could participate.

The anonymous online survey was hosted on SurveyMonkey and made available via email to GPs between July 2023 and October 2024. Data collection was finished in Germany and Switzerland in November 2023 and in Austria in October 2024. PRACTITIONER’s target sample size of at least 20 GPs per country was reached. For this substudy, we analysed the 185 questionnaires that were completed by GPs practicing in Austria, Germany and Switzerland.

### Questionnaire

The original PRACTITIONER questionnaire included only female vignettes and was piloted in two steps: first with four GPs and one cardiologist in Switzerland, and second with the national coordinators of each participating country, as described in online supplemental e appendix 1. For the German-language version, we also created otherwise identical vignettes with a male patient (‘Mr. A.’ instead of ‘Mrs. A.’), with all other clinical content kept identical; this adaptation was not independently re-piloted. The final electronic questionnaire was pilot tested on SurveyMonkey, including its survey flow, conditional logic and mandatory-item settings. Testing across different devices or browsers was not systematically documented.

Participants were randomly assigned to receive either the male or female version of the vignettes based on their date of birth (even date: female; odd date: male).

The questionnaire was 10–16 pages long, with 1–10 items per page. The number of items varied because questions about the GP’s approach to deprescription only appeared if the participant indicated they would deprescribe. Based on the results of the pilot, we informed participants that the questionnaire should take up to 10 min to complete. Participants could step backwards in the survey to change their answers but were not able to review their answers at the end of the process. If participants did not answer demographic questions or respond to the case vignettes, they could not proceed with the survey. Estimation-based demographic items and items in the third section addressing reasons for deprescribing were optional. No additional automated internal-consistency checks were applied.

The three parts of the online questionnaire comprised (online supplemental e appendix 2): (1) questions to collect GP characteristics, including sociodemographic information and practice-related variables; (2) three clinical case vignettes that described patients whose MIs had occurred recently or further in the past and who had received beta blocker therapy, with or without adverse effects; (3) questions that explored potential barriers and facilitators of beta blocker discontinuation. In this third section, participants could rate the importance of potential factors on a 5-point Likert scale.

SurveyMonkey’s multiple-response prevention setting was used to prevent more than one submission from the same browser by means of cookies. Further technical information about cookie placement and validity duration was unavailable. No registration or login was required. SurveyMonkey’s anonymous response setting was used; IP addresses and other response metadata were not collected. We followed the recommendations of the *Checklist for Reporting Results of Internet E-Surveys (CHERRIES)* (see online supplemental material).^28^

### Patient and public involvement

Patients and members of the public were not involved in the design, conduct, reporting or dissemination of this study. As the study investigated GPs’ prescribing decisions using standardised case vignettes, the questionnaire was developed and pilot tested with practising GPs, cardiologists and academic primary care researchers to ensure clinical relevance, clarity and face validity.

### Statistical analysis

We used descriptive statistics to describe GP characteristics. To assess differences in GPs’ willingness to deprescribe beta blockers across the vignette scenarios (differing in patient sex, presence/absence of side effects, time since MI), we calculated crude proportions and 95% CIs for GPs’ decisions to deprescribe for each vignette. To determine which factors influenced GPs’ decisions to deprescribe, we used generalised estimating equations with a Poisson distribution with a log link function and robust SEs, and adjusted for the clustering at the GP level, time since MI, presence of side effects, sex of the patient in the vignette, GP gender, country of practice and GP experience. We used Germany as the reference country because it had the largest sample size. We descriptively analysed GPs’ responses to items assessing reasons for deprescribing (Likert-scales). We used complete case analysis, including all non-missing responses for each analysis. RStudio (V.2024.9) was used for all statistical analyses.

## Results

### Demographics

A total of 189 respondents started the questionnaire. Of these, 172 completed the final (optional) questionnaire page, corresponding to a CHERRIES completion rate of 91.0% (172/189). Four respondents did not complete the allocation question and were excluded from the main analyses. The remaining 185 respondents completed the mandatory allocation and case-vignette items and were included in the main analyses, comprising 60 participants from Austria, 66 from Germany and 59 from Switzerland.

Baseline characteristics of GPs are shown in table 1: 35.7% (n=66) were women. 87 (47.1%) GPs were assigned to the female case vignettes and 98 (52.9%) GPs the male versions. GPs who answered questions related to case vignettes of women patients were older (mean age 52.5 years (SD 9.9)) and had worked more years (mean experience 16.5 years (SD 10.3)) than GPs who answered questions related to case vignettes of male patients (mean age 49.4 years (SD 9.6); mean work experience 14.3 years (SD 9.7)). 69.8% (n=129) of GPs conducted >25 consultations per day and 61.6% (n=114) worked in a group practice with two or more GPs. GPs overall self-estimated the prevalence of female patients at their own practices was 56.7% (SD 7.32).

**Table 1:** Characteristics of participating GPs

		Female case vignettes (n=87)	Male case vignettes (n=98)	Total (n=185)
GP age (years)		52.5 (9.9)	49.4 (9.6)	50.7 (9.8)
Work experience (years)		16.5 (10.3)	14.3 (9.7)	15.3 (10.0)
Country	Switzerland	31 (35.6)	28 (28.6)	59 (31.9)
	Germany	32 (36.8)	34 (34.7)	66 (35.7)
	Austria	24 (27.6)	36 (36.7)	60 (32.4)
GP gender	Woman	32 (36.8)	34 (34.7)	66 (35.7)
	Man	55 (63.2)	63 (64.3)	118 (63.8)
Average consultations	<15	4 (4.6)	4 (4.1)	8 (4.3)
per full working day	15–25	25 (28.7)	23 (23.5)	48 (25.9)
	26–35	29 (33.3)	32 (32.7)	61 (33.0)
	>35	29 (33.3)	39 (39.8)	68 (36.8)
Location of practice	Urban	41 (47.1)	41 (41.8)	82 (44.3)
	Suburban	17 (19.5)	15 (15.3)	32 (17.3)
	Rural	29 (33.3)	42 (42.9)	71 (38.4)
Other specialty	Yes	21 (24.1)	13 (13.3)	34 (18.4)
	No	66 (75.9)	85 (86.7)	151 (81.6)
No. GPs working at practice	One single GP	33 (37.9)	38 (38.8)	71 (38.4)
	Two or more GPs	54 (62.1)	60 (61.2)	114 (61.6)
GPs’ self-estimated prevalence of female patients in GPs’ own patient cohort		56.7 (7.9)	56.8 (6.8)	56.7 (7.32)
GPs’ self-estimated prevalence of patients with a beta blocker in their own patient cohort		21.2 (13.8)	19.4 (15.1)	20.2 (14.5)
GPs’ self-estimated prevalence of patients >65 years in their own patient cohort		54.6 (16.8)	50.5 (17.8)	52.4 (17.4)

Continuous variables are presented as mean (SD), categorical variables as number (percentage).

Missing values were n=1 (0.5%) for GP gender, GPs’ self-estimated prevalence of female patients and patients with a beta blocker in their own patient cohort and n=2 (1.1%) for GP’s self-estimated prevalence of patients >65 years.

GP, general practitioner.

### GPs’ willingness to deprescribe beta blockers according to patient sex and clinical context

GPs’ willingness to deprescribe beta blockers by patient sex and presence of side effects is shown in figure 1. In Vignette 1 (3 months after MI) without side effects, 31.0% of GPs (n=27) deprescribed beta blockers for female patients and 23.5% for male patients (n=23). In Vignette 2 (5 years after MI) without side effects, 46.0% of GPs deprescribed beta blockers for female patients (n=40) and 46.9% for male patients (n=46). In Vignette 3 (10 years after MI) without side effects, 73.6% of GPs deprescribed beta blockers for female patients (n=64) and 74.5% for male patients (n=73). When side effects were present, a much higher proportion of GPs opted to deprescribe across all vignettes. In Vignette 1, 79.3% deprescribed for the female patient (n=69) and 63.3% for the male (n=62). In Vignette 2, 83.9% deprescribed for the female patient (n=73) and 74.5% for the male one (n=73). Vignette 3 showed the highest rates of deprescription with side effects: 88.5% deprescribed for the female vignette (n=77) and 86.7% for the male one (n=85).

**Figure 1:**
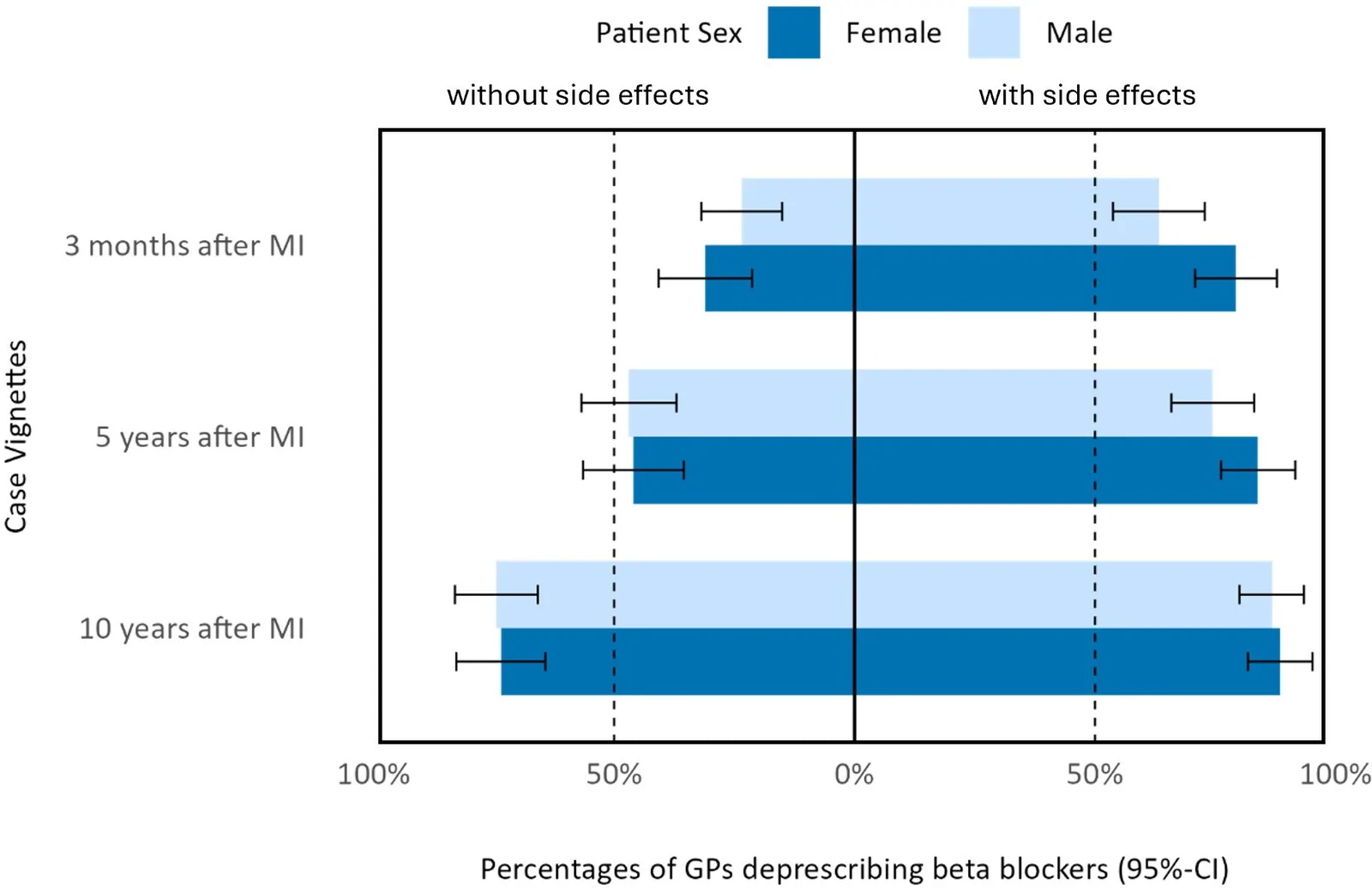
Crude percentages and 95% CIs of GPs willing to deprescribe, categorised by case vignette and patient sex. Total n for female case vignettes=87, total n for male case vignettes=98. CI (shown by bar). GPs, general practitioners; MI, myocardial infarction.

### Regression analysis

When we used 3 months post-MI as the reference, the adjusted risk ratio (RR) for deprescribing was 1.3 (95% CI 1.2 to 1.5) at 5 years and 1.7 (95% CI 1.6 to 1.9) at 10 years post-MI, as shown in table 2. The presence of side effects was associated with higher likelihood of deprescribing (RR=1.6, 95% CI 1.5 to 1.8). GPs practising in Switzerland were significantly more likely to deprescribe than GPs in Germany (RR=1.2, 95% CI 1.1 to 1.4). GPs were slightly less likely to deprescribe for male patients than female patients (RR=0.9, 95% CI 0.8 to 1.0). Work experience was not significantly associated with the willingness to deprescribe.

**Table 2:** Association between GP and characteristics of case vignettes with GPs’ willingness to deprescribe beta blockers (n=185)

Variable	Risk ratio	95% CI lower	95% CI upper	P value
Time since MI (reference: 3 months since MI)				
5 years since MI	1.3	1.2	1.5	<0.01
10 years since MI	1.7	1.6	1.9	<0.01
Presence of side effects (reference: no side effects)	1.6	1.5	1.8	<0.01
Patient sex/GP gender (reference: female/woman)				
Vignette sex=male	0.9	0.8	1.0	0.04
GP gender=man	1.1	0.9	1.2	0.30
Work experience (reference: low—quartile 1)				
Moderate (quartile 2)	1.1	0.9	1.2	0.28
High (quartile 3)	0.9	0.8	1.1	0.40
Highest (quartile 4)	1.0	0.9	1.2	0.80
Country (reference: Germany)				
Austria	1.1	1.0	1.3	0.08
Switzerland	1.2	1.1	1.4	<0.01

Adjusted for clustering at the GP level, countries added as covariates to the model. Of 1110 expected vignette responses, 14 (1.3%) were missing.

GP, general practitioner; MI, myocardial infarction.

### Reasons to deprescribe

Figure 2 illustrates the reasons GPs cited for deprescribing beta blockers after MI, categorised by the sex of the patient in the case vignettes. The most cited reason was the patient’s quality of life; 98.7% (n=78) of GPs considered this important in the female vignettes and 96.7% (n=88) in the male vignettes. For the vignettes with female patients, 97.4% (n=76) of GPs rated the perceived benefits and harms of discontinuing beta blockers as important and 92.4% (n=73) of GPs rated the perceived benefits and harms of continuing as important. For the vignettes with male patients, 85.7% (n=78) of GPs rated the perceived benefits and harms of discontinuing as important and 87.1% (n=81) rated the perceived benefits and harms of continuing as important. For the vignettes with female patients, 84.8% (n=67) of GPs cited lack of clear indication for continued beta blocker use as important; 87% (n=80) did so for the male vignettes. GPs also identified specialists’ recommendations, existence of guidelines, patients’ preferences and previous experience as important factors, listed here in descending order of importance. Overall, the same factors were most frequently cited across male and female case vignettes. The reasons reported by GPs as important for deprescribing beta blockers, stratified by patient sex, are also presented in online supplemental e appendix 3.

**Figure 2:**
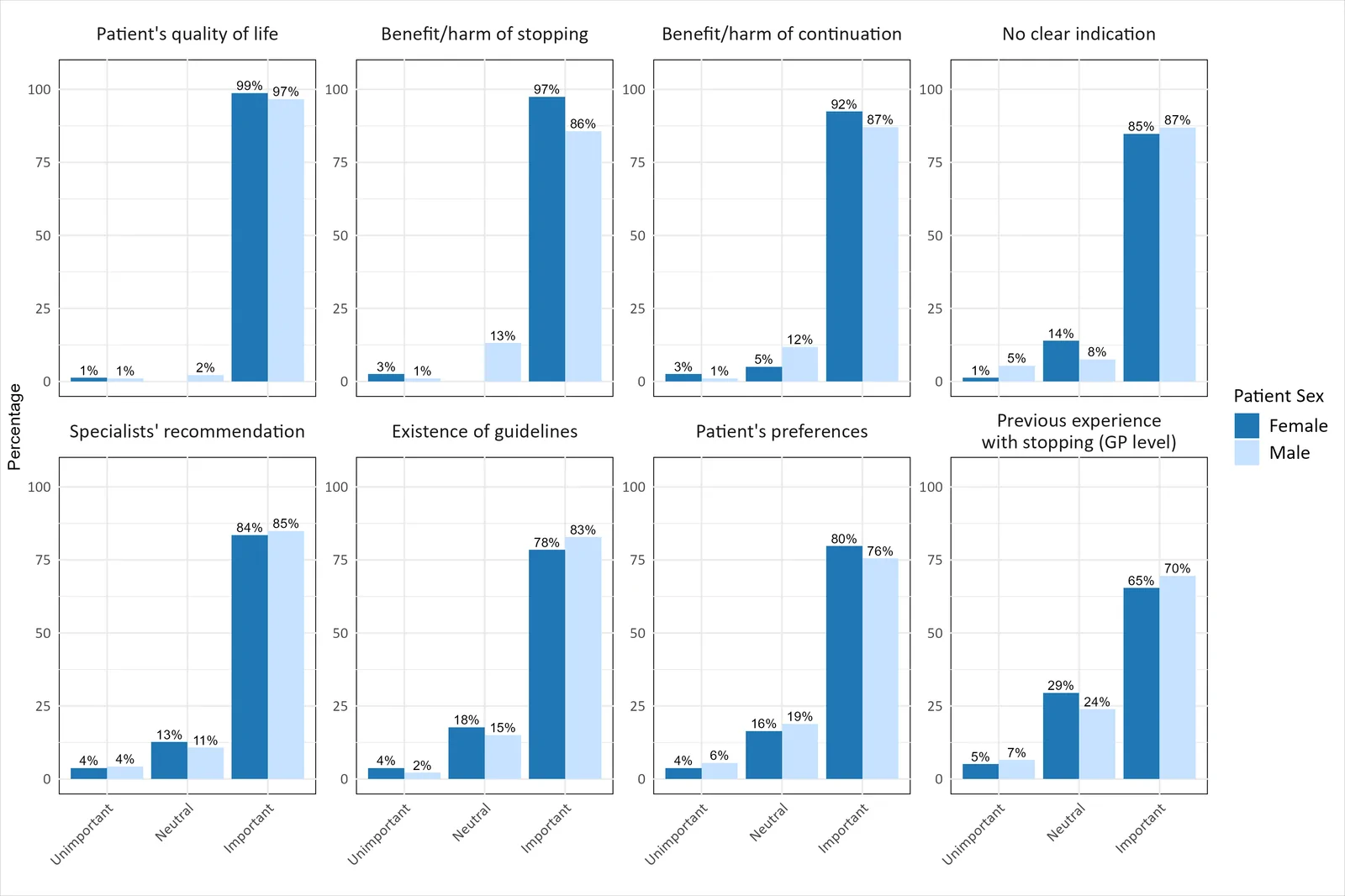
Importance of other factors influencing the willingness to deprescribe beta blockers. Percentages are based on non-missing responses. Missing responses varied slightly across items, ranging from 8 to 9 in the female vignette group (n=87) and 5 to 8 in the male vignette group (n=98). GP, general practitioner.

## Discussion

GPs’ reported willingness to deprescribe long-term beta blockers after MI with preserved LVEF varied primarily with clinical context, and male patient sex was associated with a slightly lower likelihood of GP reported deprescribing. Reported reasons for deprescribing, including considerations of quality of life and the perceived balance of benefits and harms, were similar for male and female patients.

These findings contrast with previous studies reporting sex differences in cardiovascular care. A systematic review showed that women are consistently less likely to receive evidence-based cardiovascular drugs^29^ while real-world data from Spain indicate that women are less frequently initiated on secondary prevention therapies and more often discontinued from them due to adverse effects.^30^ Biological differences, such as higher risk of adverse drug reactions in women, may further contribute to these patterns.^21^ Earlier studies had already described similar inequalities in acute coronary syndromes, where women were less likely than men to receive guideline-recommended drugs.^23 24^

One possible explanation for this discrepancy is that, in the context of long-term beta blocker therapy after MI with preserved left ventricular function, clinical decision-making occurs in an area with limited and evolving evidence, where physicians may recognise the relevance of sex and gender but lack clear guidance to incorporate these factors into practice.^31^

Beyond patient characteristics, we also explored whether physician characteristics influenced deprescribing decisions and found no evidence that GP gender influenced the decision to deprescribe. The role of physician gender in deprescribing has received little attention in the literature. Evidence from other prescribing contexts suggests that differences between male and female GPs may be small overall. For example, a study on antidepressant prescribing found few differences in treatment strategies between male and female GPs.^32^ Although a study from the in-patient setting has reported lower mortality and readmission rates among patients treated by female rather than male internists,^33^ differences associated with GP gender in our study were small compared with the influence of clinical factors such as time since MI and side effects.

Taken together, these findings suggest that clinical context may be more influential than both patient sex and physician gender in shaping deprescribing decisions for beta blockers. GPs’ willingness to deprescribe varied across vignette scenarios and was primarily driven by clinical factors, particularly time since MI and the presence of side effects, as also observed in the main PRACTITIONER study.^27^ These decisions were made in a context of limited and evolving evidence regarding the benefits of long-term beta blocker therapy after MI with preserved LVEF. Since our survey was conducted in 2023–2024, several randomised trials and subsequent pooled evidence have further informed this question.^7 8 34–36^ In particular, the individual-patient-data meta-analysis by Kristensen *et al*^37^ published after completion of our data collection, found no overall reduction in death, MI or heart failure with beta blocker therapy in patients with an LVEF ≥50%.

In the absence of clear guidance on deprescribing long-term beta blocker therapy after MI, there is even less clarity on how potential sex-specific differences should be considered in decision-making. In the meta-analysis by Kristensen *et al*, subgroup analyses did not provide clear evidence of benefit in either women or men, with a somewhat higher and less precise effect estimate among women.^37^ An observational study from Canada reported that beta blocker therapy for female patients after MI provided no clear advantage.^38^ Taken together, evidence regarding sex-specific treatment effects remains limited. Rather than pointing to systematic sex-based differences, our findings suggest that GPs approach long-term beta blocker therapy after MI with preserved left ventricular function as a context-dependent decision. At the same time, our results do not allow conclusions about the appropriateness of these decisions in real-world practice or their impact on patient outcomes.

Our study has several limitations. First, the sample size limits the generalisability of our findings even within German-speaking regions and these results should be considered exploratory. Second, we gathered a convenience sample that was voluntarily recruited through professional networks, so we cannot rule out selection bias. GPs with a stronger interest in deprescribing may have been more likely to participate, potentially leading to an overestimation of the willingness of the average GP to discontinue therapy. Third, we chose the vignette design to allow us to standardise comparison across scenarios. However, decisions in hypothetical scenarios do not necessarily reflect real-life practice. Moreover, although the survey was framed neutrally, we cannot exclude that the repeated presentation of decisions regarding beta blocker discontinuation influenced participants’ responses. The longitudinal vignette design did not allow us to disentangle the independent effects of time since MI, increasing age and, in the final vignette, increasing frailty and comorbidity. These characteristics were, however, held constant between the male and female versions of each vignette, allowing comparison according to patient sex within otherwise identical clinical scenarios. Fourth, data were collected between 2023 and 2024, before several recent trials and subsequent pooled evidence on long-term beta-blocker therapy after MI were published. The findings therefore reflect decision-making in the context of the evidence available at the time and may not fully represent current clinical practice.

## Conclusion

Patient sex showed a small association with GPs’ reported willingness to deprescribe long-term beta blockers after MI with preserved left ventricular function. In contrast, willingness to deprescribe varied substantially across clinical scenarios and was higher with longer time since MI and in the presence of side effects, suggesting that GPs weigh both the evolving benefit-risk balance over time and the immediate burden of treatment in their decision-making. Overall, these findings suggest that clinical context plays a greater role than patient sex in GPs’ reported deprescribing decisions.

## Supplementary material

10.1136/bmjopen-2026-125086 online supplemental file 1

10.1136/bmjopen-2026-125086 online supplemental file 2

## Data Availability

Data are available upon reasonable request.
