# Supplementary material for: Role of patient sex in prescribing long-term beta blockers after myocardial infarction in German-speaking European primary care settings: a cross-sectional online survey using case vignettes
Source: BMJ Open. 2026 Oct 23;16(10):e125086. doi: 10.1136/bmjopen-2026-125086 (PMC13711600; doi:10.1136/bmjopen-2026-125086)
Supplement: Supplementary file 1 [file bmjopen-2026-125086.reviewer_comments.pdf]

PEER REVIEW HISTORY

BMJ Open publishes all reviews undertaken for accepted manuscripts. Reviewers are asked to complete a checklist review form and are provided with free text boxes to elaborate on their assessment. These free text comments are reproduced below.

ARTICLE DETAILS

Title (Provisional)

THE ROLE OF PATIENT SEX IN PRESCRIBING LONG-TERM BETA BLOCKERS AFTER MYOCARDIAL INFARCTION IN GERMAN-SPEAKING EUROPEAN PRIMARY CARE SETTINGS – A CROSS-SECTIONAL ONLINE SURVEY USING CASE VIGNETTES

Authors

Grindat, Rahel; Jungo, Katharina T; Tal, Kali; Brütting, Christine; Erber, Patrick; Moussa, Mahmoud; Zelko, Erika; Streit, Sven; Zangger, Martina

---

VERSION 1 - REVIEW

**Reviewer**	**1**
**Name**	**Kohno, Takashi**
**Affiliation**	**Keio**
**Date**	**23-Aug-2026**
**COI**	**None**

The authors conclude that, based on this cross-sectional online survey using case vignettes, the decision to deprescribe long-term beta-blockers was primarily associated with the time elapsed since myocardial infarction and the presence of side effects, with patient gender showing only a modest association. However, major concern regarding novelty and interpretation of the main findings.

The main findings of this study appear to substantially overlap with those previously reported in Reference 25 (BMC Prim Care. 2026;27:93.).

The finding that patient gender showed only a small association with deprescribing decisions seems insufficient, in our view, to establish sufficient novelty to justify publication as a separate manuscript.

The authors conclude that “GPs’ decisions to deprescribe long-term beta blockers were primarily influenced by time since myocardial infarction.” However, it remains unclear whether this association reflects the time elapsed since myocardial infarction itself or

differences in patient age and comorbidities that are likely to accompany a longer interval since myocardial infarction.

More importantly, age and comorbidity burden are clinically important determinants of beta-blocker continuation or deprescribing in their own right and may represent more fundamental factors for understanding deprescribing decisions than the relatively small association observed with patient gender. A more detailed assessment of these factors would therefore be necessary before focusing on the role of gender.

The evidence in this topic has continued to evolve, and the relevant literature should be cited and discussed in greater detail. Furthermore, the timing of the survey is important. Depending on when the data were collected, the reported prescribing and deprescribing patterns may not adequately reflect current clinical practice and contemporary evidence.

There are also important concerns regarding the robustness of the findings. The relatively small sample size may result in unstable estimates. The significant difference in the distribution of “other specialties” between the groups raises the possibility that specialty-specific practice patterns may have contributed to the observed differences. This potential confounding should be carefully addressed.

**Reviewer**	**2**
**Name**	**Tobe, Sheldon**
**Affiliation**	**Sunnybrook Health Sciences Centre, Medicine**
**Date**	**25-Aug-2026**
**COI**	**None**

The study reports on GPs’ willingness to deprescribe beta blockers in patients post MI who have preserved left ventricular function from their response to an online survey using case vignettes. The vignettes differed by patient’s sex, time since MI, and the presence or absence of side effects. It also explored whether the participant’s gender influenced their willingness to deprescribe. It found a greater willingness to deprescribe beta blockers with longer duration from the MI, and side effects from the beta blockers. There was a slight decrease in deprescribing for male patients, and no difference based on the reported sex of the participants.

This report is a cross sectional substudy of the PRACTITIONER study, looking only at questionnaires from three of the 20 countries which had vignettes of both male and female patients, while many of the other countries had only vignettes of female patients.

This report is problematic for me for a number of reasons.

1. The way that gender and sex are used in the report.

2. The implication in the survey which is focused on deprescribing beta blockers post MI given to the GPs who participated in the survey, and the balance of the literature which remains mixed on the subject.

1. Gender and Sex: The Canadian Institutes of Health Research (CIHR) defines sex and gender as follows:

Sex: “Sex refers to a set of biological attributes in humans and animals. It is primarily associated with physical and physiological features including chromosomes, gene expression, hormone levels and function, and reproductive/sexual anatomy. Sex is usually categorized as female or male but there is variation in the biological attributes that comprise sex and how those attributes are expressed.” [cihr-irsc.gc.ca]

Gender: “Gender refers to the socially constructed roles, behaviours, expressions and identities of girls, women, boys, men, and gender diverse people. It influences how people perceive themselves and each other, how they act and interact, and the distribution of power and resources in society. Gender identity is not confined to a binary (girl/woman, boy/man) nor is it static; it exists along a continuum and can change over time.” [cihr-irsc.gc.ca]

While referring to Gender, the GPs can define themselves as Man, Woman, or Non-binary person. The results are only presented as either man or woman, or male female. Table 1 lists GP Gender as woman or man. Table 2 Lists Gender reference: female, and Vignette gender = male, and GP gender = male. Also the data is presented in Figure 1 as Patient Gender female and male.

The reporting of sex and gender in the manuscript would benefit from more consistency and reviewing the BMJ editorial policy 1.

2. Regarding the message given to GP’s participating in the survey about deprescribing beta blockers, the survey includes this sentence in the welcome paragraph: ‘As there is little recent evidence on this topic, the optimal treatment regarding beta blockers is unclear . We would like to find out which factors influence your decision to prescribe beta blockers or not and how prescriptions differ between different regions in Europe.’

Until I read this article, I was not aware of the mass of new evidence on beta blocker deprescribing post MI, and the intense efforts underway to resolve the issue so it seems likely to me that the GPs were also largely unaware. This information is not available from the survey. The fact that all the cases dealt with deprescribing beta blockers sends the message that this topic is of importance. Without clear directions in recent clinical practice guidelines, GPs taking the survey may have been puzzled about why they were being asked these questions. This could have been a factor that could sway participants to be more assertive in the questionnaire about deprescribing then they were in actual practice.

Introduction:

References 11 to 13 are cited to reflect inconsistent advice on the length of beta blocker therapy post MI. More recent guidelines also support this issue and should be considered for addition: The 2023 ESC guideline states: Routine beta-blockers for all ACS patients regardless of LVEF should be considered 2. The 2025 ACC/AHA guideline for the management of acute coronary syndromes states that 'The optimal duration of beta blocker use remains unclear in patients with preserved LVEF' 3. Also note that The Silvain reference is incomplete (reference 7) and the 2023 ESC guidelines reference 33. Reference 34 should be a systematic review, and not the RCT reported by Munkhaugen.

Page 6 Line 54, The Sentence The PRACTITIONER study, should be clarified to refer to the publication on the PRACTITIONER study, otherwise it is confusing to read the sentence below on page 7 line 5.

The methods are otherwise clear and concise.

Results: Page 10 Line 45. This paragraph would be easier to understand if it was made into a table.

Discussion: The paragraph on page 12 line 39 to the end includes recent literature on beta blocker continuation post MI.

Some of the following might also benefit the discussion also as recent additions to the literature:

Trials of not using BB post MI with preserved EF

• Munkhaugen J – BETAMI-DANBLOCK investigators. NEJM 2025. 4

• Yndigegn T NEJM 2024 5

• Ibanez B, NEJM 2025 6

Studies of Discontinuation

• Silvain J Beta blocker interruption vs continuation post MI. NEJM 2024: 7

• Choi KH NEJM 2026: 8

1. Peters SAE, Norton R. Sex and gender reporting in global health: new editorial policies. BMJ Global Health 2018;3(4):e001038. doi: 10.1136/bmjgh-2018-001038

2. Byrne R, Rossello X, Coughlan J, et al. 2023 ESC guidelines for the management of acute coronary syndromes. COR ET VASA 2024;66(2):170–232.

3. Rao SV, O'Donoghue ML, Ruel M, et al. 2025 ACC/AHA/ACEP/NAEMSP/SCAI Guideline for the Management of Patients With Acute Coronary Syndromes: A Report of the American College of Cardiology/American Heart Association Joint Committee on Clinical Practice Guidelines. Circulation 2025;151(13):e771–e862. doi: 10.1161/cir.0000000000001309 [published Online First: 20250227]

4. Munkhaugen J, Kristensen AMD, Halvorsen S, et al. Beta-blockers after myocardial infarction in patients without heart failure. New England Journal of Medicine 2025;393(19):1901–11. doi: DOI: 10.1056/NEJMoa2505985

5. Yndigegn T, Lindahl B, Mars K, et al. Beta-Blockers after Myocardial Infarction and Preserved Ejection Fraction. N Engl J Med 2024;390(15):1372–81. doi: 10.1056/NEJMoa2401479 [published Online First: 20240407]

6. Ibanez B, Latini R, Rossello X, et al. Beta-blockers after myocardial infarction without reduced ejection fraction. New England Journal of Medicine 2025;393(19):1889–900.

7. Silvain J, Cayla G, Ferrari E, et al. Beta-Blocker Interruption or Continuation after Myocardial Infarction. The New England journal of medicine 2024;391(14):1277–86. doi: 10.1056/NEJMoa2404204

8. Choi KH, Kang D, Kim W, et al. Discontinuation of Beta-Blocker Therapy after Myocardial Infarction. The New England journal of medicine 2026;394(13):1302–12. doi: 10.1056/NEJMoa2601005

VERSION 1 - AUTHOR RESPONSE

Reviewer 1

1. The main findings of this study appear to substantially overlap with those previously reported in Reference 25 (BMC Prim Care. 2026;27:93.). The finding that patient gender showed only a small association with deprescribing decisions seems insufficient, in our view, to establish sufficient novelty to justify publication as a separate manuscript.

Response 1: Please see our combined response to Editorial Comment 2 and Reviewer 1, Comment 1 above

2. More importantly, age and comorbidity burden are clinically important determinants of beta-blocker continuation or deprescribing in their own right and may represent more fundamental factors for understanding deprescribing decisions than the relatively small association observed with patient gender. A more detailed assessment of these factors would therefore be necessary before focusing on the role of gender.

Response 2:

We thank the reviewer for this important observation and agree that age, frailty, and comorbidity burden are clinically important determinants of deprescribing decisions. These factors formed part of the broader clinical context investigated in the main PRACTITIONER study (Reference 25), which examined determinants of physicians' willingness to deprescribe long-term beta blockers. The specific aim of the present substudy, however, was not to reassess the relative contribution of these clinical determinants, but to examine whether patient sex was additionally associated with deprescribing decisions.

We agree that the vignette design does not allow the independent effects of time since myocardial infarction, age, and increasing clinical complexity to be disentangled. The three

vignettes describe the same patient longitudinally: age increases alongside time since myocardial infarction, and the third vignette additionally introduces frailty and comorbidity. Importantly, however, these clinical characteristics were identical between the male and female versions of each vignette; only the patient's sex differed. Thus, while our design cannot determine the independent contributions of these clinical factors, they do not account for differences between the male and female vignette groups.

We have now explicitly acknowledged this limitation in the Discussion, while clarifying that the standardized clinical characteristics across the male and female vignette versions allow the comparison according to patient sex within otherwise identical clinical scenarios (p. 12)

«The longitudinal vignette design did not allow us to disentangle the independent effects of time since myocardial infarction, increasing age, and, in the final vignette, increasing frailty and comorbidity. These characteristics were, however, held constant between the male and female versions of each vignette, allowing comparison according to patient sex within otherwise identical clinical scenarios"

3. The evidence in this topic has continued to evolve, and the relevant literature should be cited and discussed in greater detail. Furthermore, the timing of the survey is important. Depending on when the data were collected, the reported prescribing and deprescribing patterns may not adequately reflect current clinical practice and contemporary evidence.

Response 3: We thank the reviewer for this important comment and agree that the timing of data collection is relevant when interpreting our findings. Data were collected between July 2023 and October 2024; recruitment in Switzerland and Germany was completed by November 2023, while recruitment in Austria continued until October 2024. Several major trials that have subsequently contributed to the evidence on long-term beta-blocker therapy after myocardial infarction were therefore published either during or after data collection. Importantly, the meta-analysis by Kristensen et al., which synthesized evidence from several recent randomized trials, was not published until November 2025, more than a year after data collection had ended. Our findings should therefore be interpreted as reflecting GPs' decision-making in the context of the evidence and guideline recommendations available at the time of the survey, rather than current clinical practice in 2026.

We have therefore updated the Discussion to reflect the evidence that has emerged since the survey was conducted. The revised Discussion now states (p. 11-12):

"These decisions were made in a context of limited and evolving evidence regarding the benefits of long-term beta blocker therapy after myocardial infarction with preserved LVEF. Since our survey was conducted in 2023–2024, several randomized trials and subsequent pooled evidence have further informed this question [7,34–36]. In particular, the individual-patient-data meta-analysis by Kristensen et al. [37], published after completion of our data collection, found no overall reduction in death, myocardial infarction, or heart failure with beta blocker therapy in patients with an LVEF ≥50%."

We have also added the timing of data collection as a limitation (p. 12):

“Fourth, data were collected between 2023 and 2024, before several recent trials and subsequent pooled evidence on long-term beta-blocker therapy after myocardial infarction were published. The findings therefore reflect decision-making in the context of the evidence available at the time and may not fully represent current clinical practice.”

4. There are also important concerns regarding the robustness of the findings. The relatively small sample size may result in unstable estimates.

Response 4: We agree with the reviewer that the relatively small sample size is an important limitation. This substudy used the available German-language subsample from the broader PRACTITIONER study; no independent a priori sample size calculation was performed for this sex-focused research question. The analysis should therefore be interpreted as exploratory rather than confirmatory. We have slightly adapted the limitations section to make this explicit (p.12).

“First, the sample size limits the generalizability of our findings even within German-speaking regions and these results should be considered exploratory.”

5. The significant difference in the distribution of “other specialties” between the groups raises the possibility that specialty-specific practice patterns may have contributed to the observed differences. This potential confounding should be carefully addressed.

Response 5: We thank the reviewer for raising this point. Upon re-verifying our dataset in response to this comment, we identified a labeling error in Table 1: the “No” and “Yes” categories for “other specialty” had been transposed. We have corrected this error. The accurate distribution shows that only a minority of GPs held an additional specialty besides general practice (female-vignette group: 24.1%, n=21; male-vignette group: 13.3%, n=13). Thus, the difference noted by the reviewer concerned a relatively small subgroup of participants rather than the majority of GPs in either vignette group. We have corrected Table 1 accordingly and thank the reviewer for drawing our attention to this error.

Reviewer 2

1. While referring to Gender, the GPs can define themselves as Man, Woman, or Non-binary person. The results are only presented as either man or woman, or male female. Table 1 lists GP Gender as woman or man. Table 2 Lists Gender reference: female, and Vignette gender = male, and GP gender = male. Also the data is presented in Figure 1 as Patient Gender female and male. The reporting of sex and gender in the manuscript would benefit from more consistency and reviewing the BMJ editorial policy 1.

Response 1: We thank the reviewer for this important observation and fully agree that the reporting of sex and gender in our manuscript warrants greater consistency, in line with the BMJ editorial policy on sex and gender reporting.

GPs' gender identity was assessed using three response options ("Woman" / "Man" / "Non-binary person"; Supplement, eAppendix 2), no participant identified as non-binary. Regarding the case vignettes, we agree that "sex" is the more accurate term, as this reflects a fixed biological characteristic assigned to the hypothetical patient rather than a self-identified gender. We would also note that the original questionnaire was administered in German, a language that does not distinguish linguistically between "sex" and "gender" (both are captured by the single term "Geschlecht").

We have revised the manuscript throughout to reflect this distinction consistently: the title, Table 1, Table 2, Figure 1, and the relevant passages in the Abstract, Methods, Results and Discussion now use "sex" for the patient/vignette variable and "gender" for GPs' self-reported gender identity.

2. Without clear directions in recent clinical practice guidelines, GPs taking the survey may have been puzzled about why they were being asked these questions. This could have been a factor that could sway participants to be more assertive in the questionnaire about deprescribing then they were in actual practice.

Response 2: We thank the reviewer for this consideration. As already described in the Supplement (eAppendix 1), the survey was deliberately framed broadly as investigating decision-making, using a neutral title that did not refer to deprescribing, in order to minimize this risk. Nevertheless, we agree that repeatedly presenting decisions regarding beta blocker discontinuation may have influenced participants' responses. We have therefore explicitly acknowledged this possibility in the limitations of the revised manuscript (p. 12d):

"Moreover, although the survey was framed neutrally, we cannot exclude that the repeated presentation of decisions regarding beta blocker discontinuation influenced participants' responses."

3. References 11 to 13 are cited to reflect inconsistent advice on the length of beta blocker therapy post MI. More recent guidelines also support this issue and should be considered for addition: The 2023 ESC guideline states: Routine beta-blockers for all ACS patients regardless of LVEF should be considered 2. The 2025 ACC/AHA guideline for the management of acute coronary syndromes states that 'The optimal duration of beta blocker use remains unclear in patients with preserved LVEF' 3.

Response 3: We thank the reviewer for this suggestion. We have updated the Introduction to explicitly incorporate the recommendations from the 2023 ESC and 2025 ACC/AHA guidelines. The revised Introduction now states (p. 5):

"The 2023 ESC guidelines recommend considering routine beta blocker therapy after acute coronary syndrome regardless of LVEF, whereas the 2025 ACC/AHA guideline states that the optimal duration of beta blocker therapy remains unclear in patients with preserved LVEF [15,16]."

4. Also note that The Silvain reference is incomplete (reference 7) and the 2023 ESC guidelines reference 33.

Response 4: We thank the reviewer for pointing out these reference issues. We have completed the bibliographic information for the Silvain et al. reference and corrected the reference to the 2023 ESC guidelines. The reference list has been updated accordingly.

5. Reference 34 should be a systematic review, and not the RCT reported by Munkhaugen.

Response 5: We thank the reviewer for identifying this citation error. The statement was intended to refer to the individual-patient-data meta-analysis by Kristensen et al., rather than the randomized trial by Munkhaugen et al. We have corrected the citation and revised the text to more accurately reflect the subgroup findings.

6. Page 6 Line 54, The Sentence The PRACTITIONER study, should be clarified to refer to the publication on the PRACTITIONER study, otherwise it is confusing to read the sentence below on page 7 line 5.

Response 6: We thank the reviewer for pointing out this ambiguity. We have revised the Introduction to distinguish more clearly between the overarching PRACTITIONER study and the publication reporting its main findings. We have also clarified that the previous publication investigated factors associated with GPs' willingness to deprescribe long-term beta blockers but did not examine patient sex, and that the present comparison was possible only in Austria, Germany, and Switzerland, where otherwise identical male and female vignette versions were administered. The introduction now reads (p. 6):

"The publication reporting the PRACTITIONER study investigated factors associated with GPs' willingness to deprescribe long-term beta blockers after myocardial infarction but did not examine the role of patient sex [27]. A comparison according to patient sex was possible only in Austria, Germany, and Switzerland, where otherwise identical male and female versions of the case vignettes were administered. In this study, we therefore investigated whether patient sex was associated with GPs' willingness to deprescribe beta blockers in this clinical context."

7. Results: Page 10 Line 45. This paragraph would be easier to understand if it was made into a table.

Response 7: We thank the reviewer for this suggestion and agree that a tabular presentation can facilitate interpretation of the detailed results. As Figure 2 already provides a visual comparison of these factors by patient sex, we preferred to retain the figure in the main manuscript to avoid presenting the same data twice. We have therefore added a table with the detailed percentages and frequencies for each factor, by patient sex, to the Supplement (eAppendix 3).

Table 3: Reasons cited by GPs as important for deprescribing beta blockers, by patient sex.

Factor	Female vignettes (n)	Male vignettes (n)
Patient's quality of life	98.7% (78)	96.7% (88)
Benefits / harms of stopping	97.4% (76)	85.7% (78)
Benefits / harms of continuation	92.4% (73)	87.1% (81)
No clear indication	84.8% (67)	87.0% (80)
Specialists' recommendation	83.5% (66)	84.9% (79)
Existence of guidelines	78.5% (62)	82.8% (77)
Patient's preferences	79.7% (63)	75.6% (68)
Previous experience with stopping (GP level)	65.4% (51)	69.6% (64)

8. Discussion: The paragraph on page 12 line 39 to the end includes recent literature on beta blocker continuation post MI. Some of the following might also benefit the discussion also as recent additions to the literature:

Response 8: We thank the reviewer for highlighting these recent additions to the literature. Several of the studies suggested by the reviewer were already cited in the manuscript. We have now updated the Discussion to incorporate the additional recent evidence, including the studies by Ibanez et al. and Choi et al.

VERSION 2 - REVIEW

**Reviewer**	**1**
**Name**	**Kohno, Takashi**
**Affiliation**	**Keio**
**Date**	**06-Oct-2026**
**COI**	

My comments have been addressed adequately and the manuscript has improved substantially
