# Supplementary material for: Role of patient sex in prescribing long-term beta blockers after myocardial infarction in German-speaking European primary care settings: a cross-sectional online survey using case vignettes
Source: BMJ Open. 2026 Oct 23;16(10):e125086. doi: 10.1136/bmjopen-2026-125086 (PMC13711600; doi:10.1136/bmjopen-2026-125086)
Supplement: Supplementary file 3 [file bmjopen-16-10-s002.docx]

**The role of patient sex in prescribing long-term beta blockers after myocardial infarction in German-speaking European primary care settings – a cross-sectional online survey using case vignettes**

**Supplemental Material**

*Authors: Rahel Grindat, MD^1^; Katharina Tabea Jungo^1,2^, PhD; Kali Tal, PhD^1^, Christine Brütting, PhD^3^; Patrick Erber, MD^4^; Mahmoud Moussa, MD^5^; Erika Zelko, ND, PhD^6^; Sven Streit, MD, PhD, MPH^1^; Martina Zangger, MD^1,7^*

1 Institute of Primary Health Care (BIHAM), University of Bern, Bern, Switzerland
2 Center for Healthcare Delivery Science and Division of Pharmacoepidemiology and Pharmacoeconomics, Department of Medicine, Brigham and Women’s Hospital and Harvard Medical School, Boston, Massachusetts, United States of America
3 Institute of General Practice, Faculty of Medicine, University of Halle-Wittenberg, Halle (Saale), Saxony-Anhalt, Germany
4 Division General and Family Medicine, Department of General Health Studies, Karl Landsteiner University of Health Sciences, Krems, Austria
5 Department of Primary Care Medicine, Medical University of Vienna, Vienna, Austria
6 Institute for General Medicine, Johannes Kepler Universität Linz, Linz, Austria
7 Graduate Health School for Health Sciences, University of Bern, Bern, Switzerland

**Table of content**

[eAppendix 1. Questionnaire development and piloting	3](#_Toc239505061)

[eAppendix 2: Full questionnaire	4](#_Toc239505062)

[eAppendix 3: Reasons cited by GPs as important for deprescribing beta blockers, by patient sex.	9](#_Toc239505063)

# eAppendix 1. Questionnaire development and piloting

**Initial development and pilot testing**

The original PRACTITIONER questionnaire (female vignettes only) was piloted in Switzerland with four GPs and one cardiologist. This pilot informed the following adaptations:

- Implementation of conditional logic in the case vignettes (follow-up questions displayed only if deprescribing was selected)
- Explicit clarification of clinical stability (normal heart rate and blood pressure; electrocardiogram without atrioventricular block)
- Use of a neutral survey title not referring to deprescribing
- Explicit description of the presence or absence of side effects in the vignettes
- Removal of mandatory responses for estimation-based demographic items (e.g., proportion of female patients)

**Review by national coordinators**

National coordinators provided feedback on the questionnaire, including general and country-specific aspects. Two coordinators provided broader feedback beyond translation. This resulted in:

- Rephrasing of items on practice structure to ensure applicability across different healthcare systems
- Clarification on how to derive percentages from absolute numbers (e.g., number of female patients)
- Allowing the use of beta blockers other than metoprolol (e.g., bisoprolol) depending on local prescribing practices

**Gender-adapted version**

- For the German-language version, male vignettes were created by changing the patient designation from “Frau A” to “Herr A” without additional piloting.

**Country-specific adaptations**

Minor wording adaptations were made to reflect national terminology:

- Germany: modification of the question on additional specialist qualifications
- Austria: modification of the same item to reflect national certification terminology

# eAppendix 2: Full questionnaire

1. Welcome!

Thank you for your interest in our project!

We are conducting a survey to investigate general practitioner's (de)prescribing decisions with regards to beta

blocker prescriptions after uncomplicated myocardial infarction at the Institute for Primary Health Care at the

University of Bern, Switzerland.

As there is little recent evidence on this topic, the optimal treatment regarding beta blockers is unclear . We

would like to find out which factors influence your decision to prescribe beta blockers or not and how

prescriptions diﬀer between diﬀerent regions in Europe.

Participation is anonymous and all data will be treated confidentially . The survey is divided into three parts and

should not take more than 10 minutes to complete.

If you agree to participate, please press the "next " button.

Thank you for your support,

Sven Streit, Katharina Jungo and Martina Zangger on behalf of the rest of the study team

2. GP characteristics

First, let us start with a few sociodemographic questions:

1. How old are you (in number of years)?

2. What is your gender identity?

- Woman
- Man
- Non-binary person

3. How many years have you been practicing as GP (in number of years)?

4. How many clinical consultations do you have on average per full working day?

- <15
- 15-25
- 26-35
- >35

5. Where is your practice located at?

- urban
- suburban
- rural

6. Do you have another specialisation apart from GP?

- No
- Yes (please specify)

7. How many general practitioners work at your workplace (inlcuding you)?

- One single GP
- Two or more GPs

8. Make a rough estimate: What percentage of your patients is female?

(If you happen to know the absolute number of female patients in your practice divide that by the total number of patients and multiply by 100 to get the percentage)

9. Make a rough estimate: What percentage of your patients is 65 years or older?

(If you happen to know the absolute number of patients >65 years in your practice divide that by the total number of patients and multiply by 100 to get the percentage)

10. Make a rough estimate: what percentage of your patients is prescribed a beta blocker (for any indication)?

(If you happen to know the absolute number of patients taking a beta blocker in your practice divide that by the total number of patients and multiply by 100 to get the percentage)

3. Work practice

In order for us to understand your daily work practice, please answer the following questions

11. Do you regularly discuss patients with colleagues (e.g., with other GPs in your practice or as part of a quality management program)?

- Never
- Rarely (a few times a year)
- On a monthly basis
- On a weekly basis
- On daily basis

12. In the past month, have you considered discontinuing a beta blocker for any reason for any of your patients?

- Yes
- No

13. Are you aware of any guidelines that address the discontinuation of betablockers after myocardial infarction with preserved left ventricular function?

- No
- Yes, which one

14. In your experience, who starts beta blocker prescription after myocardial infarction? (tick all that apply)

- Hospital where the myocardial infarction was treated initially
- Cardiologist
- General Practitioner
- Other (please specify)

15. Who is responsible for the continuation of beta blocker prescription after myocardial infarction in your region? (tick all that apply)

- Hospital where the myocardial infarction was treated initially
- Cardiologist
- General Practitioner
- Other (please specify)

4. Case vignette 1

In the following, three hypothetical case vignettes are presented. Please specify how you would treat the patient if she came to your practice.

Your Patient, Mrs. A, is a 68-year-old woman and comes to your practice for a follow-up after she had an acute

ST -elevation myocardial infarction with placement of a single drug eluting stent three months ago.

Since then, she takes a dual antiplatelet therapy with aspirin (100mg/d) and ticagrelor (180mg/d) as well

as atorvastatin (40mg/d) and metoprolol (100mg/d) regularly . She does not take any other medication. She

has recovered well and does not suﬀer from residual symptoms such as chest pain. Her left ventricular

function is normal with no signs of heart failure or arrythmia. Otherwise, her medical history is

unremarkable, she is retired and spends most of her time looking after her grandchildren. She reports feeling

well. Her vital parameters (pulse and blood pressure) and the clinical examination are normal to you. The EKG

shows no AV-block.

16. Would you stop or reduce the dosages of her metoprolol?

- yes (stop or reduce)
- no (keep the same dosage)

5. Case vignette 1

(same text) Your Patient, Mrs. A, is a 68-year-old woman and comes to your practice for a follow-up after she

had an acute ST -elevation myocardial infarction with placement of a single drug eluting stent three

months ago. Since then, she takes a dual antiplatelet therapy with aspirin (100mg/d) and ticagrelor

(180mg/d) as well as atorvastatin (40mg/d) and metoprolol (100mg/d) regularly . She does not take any

other medication. She has recovered well and does not suﬀer from residual symptoms such as chest pain.

Her left ventricular function is normal with no signs of heart failure or arrythmia. Otherwise, her

medical history is unremarkable, she is retired and spends most of her time looking after her grandchildren.

She reports feeling well. Her vital parameters (pulse and blood pressure) and the clinical examination are

normal to you. The EKG shows no AV-block.

17. Since you replied "yes" to the previous question, how would you change the dosage?

- reduce dose
- stop medication (= 100% dose reduction, no matter if tapering or direct stop)

6. Case vignette 1

(same text) Your Patient, Mrs. A, is a 68-year-old woman and comes to your practice for a follow-up after she

had an acute ST -elevation myocardial infarction with placement of a single drug eluting stent three

months ago. Since then, she takes a dual antiplatelet therapy with aspirin (100mg/d) and ticagrelor

(180mg/d) as well as atorvastatin (40mg/d) and metoprolol (100mg/d) regularly . She does not take any

other medication. She has recovered well and does not suﬀer from residual symptoms such as chest pain.

Her left ventricular function is normal with no signs of heart failure or arrythmia. Otherwise, her

medical history is unremarkable, she is retired and spends most of her time looking after her grandchildren.

She reports feeling well. Her vital parameters (pulse and blood pressure) and the clinical examination are

normal to you. The EKG shows no AV-block.

18. Would you stop or reduce the dosages of her metoprolol if she were complaining of dizziness when standing up?

- yes (stop or reduce)
- no (keep the same dosage)

7. Case vignette 1

(same text) Your Patient, Mrs. A, is a 68-year-old woman and comes to your practice for a follow-up after she

had an acute ST -elevation myocardial infarction with placement of a single drug eluting stent three

months ago. Since then, she takes a dual antiplatelet therapy with aspirin (100mg/d) and ticagrelor

(180mg/d) as well as atorvastatin (40mg/d) and metoprolol (100mg/d) regularly . She does not take any

other medication. She has recovered well and does not suﬀer from residual symptoms such as chest pain.

Her left ventricular function is normal with no signs of heart failure or arrythmia. Otherwise, her

medical history is unremarkable, she is retired and spends most of her time looking after her grandchildren.

She reports feeling well. Her vital parameters (pulse and blood pressure) and the clinical examination are

normal to you. The EKG shows no AV-block.

19. Since you replied "yes" to the previous question, how would you change the dosage if the patient were complaining of dizziness when standing up?

- reduce dose
- stop medication (= 100% dose reduction, no matter if tapering or direct stop)8. Case vignette 2

8. Case vignette 2

The same patient, Mrs. A., comes five years later for a routine follow-up. She is now 73 years old and

continues to take aspirin (100mg/d), atorvastatin (40mg/d) and metoprolol (100mg/d) regularly . Apart

from the myocardial infarction five years ago, her medical history remained unremarkable. Her left

ventricular function is still normal with no signs of heart failure or arrythmia. She is still feeling well

and does not suﬀer from any residual symptoms. Her vital parameters (pulse and blood pressure) and the

clinical examination are normal to you. The EKG shows no AV-block.

20. Would you stop or reduce the dosages of her metoprolol?

- yes (stop or reduce)
- no (keep the same dosage)

9. Case vignette 2

(same text) The same patient, Mrs. A., comes five years later for a routine follow-up. She is now 73 years old

and continues to take aspirin (100mg/d), atorvastatin (40mg/d) and metoprolol (100mg/d) regularly .

Apart from the myocardial infarction five years ago, her medical history remained unremarkable. Her left

ventricular function is still normal with no signs of heart failure or arrythmia. She is still feeling well

and does not suﬀer from any residual symptoms. Her vital parameters (pulse and blood pressure) and the

clinical examination are normal to you. The EKG shows no AV-block.

21. Since you replied "yes" to the previous question, how would you change the dosage?

- reduce dose
- stop medication (= 100% dose reduction, no matter if tapering or direct stop)

10. Case vignette 2

(same text) The same patient, Mrs. A., comes five years later for a routine follow-up. She is now 73 years old

and continues to take aspirin (100mg/d), atorvastatin (40mg/d) and metoprolol (100mg/d) regularly .

Apart from the myocardial infarction five years ago, her medical history remained unremarkable. Her left

ventricular function is still normal with no signs of heart failure or arrythmia. She is still feeling well

and does not suﬀer from any residual symptoms. Her vital parameters (pulse and blood pressure) and the

clinical examination are normal to you. The EKG shows no AV-block.

22. Would you stop or reduce the dosages of her metoprolol if she were complaining of dizziness when standing up?

- yes (stop or reduce)
- no (keep the same dosage)

11. Case vignette 2

(same text) The same patient, Mrs. A., comes five years later for a routine follow-up. She is now 73 years old

and continues to take aspirin (100mg/d), atorvastatin (40mg/d) and metoprolol (100mg/d) regularly .

Apart from the myocardial infarction five years ago, her medical history remained unremarkable. Her left

ventricular function is still normal with no signs of heart failure or arrythmia. She is still feeling well

and does not suﬀer from any residual symptoms. Her vital parameters (pulse and blood pressure) and the

clinical examination are normal to you. The EKG shows no AV-block.

23. Since you replied "yes" to the previous question, how would you change the dosage if the patient were complaining of dizziness when standing up?

- reduce dose
- stop medication (= 100% dose reduction, no matter if tapering or direct stop)

12. Case vignette 3

Another five years later, Mrs. A, now 78 years old, is moved to a nursing home. She has been diagnosed with

Alzheimer’s disease and is strongly dependent on support for her activities of daily living such as dressing,

personal hygiene and feeding. She only hardly walks on the rollator and has lost 5kg of weight in the past

six months. She still takes her aspirin (100mg/d), atorvastatin (40mg/d) and metoprolol (100mg/d)

regularly . Her left ventricular function is still normal with no signs of heart failure or arrythmia. Her vital

parameters (pulse and blood pressure) are normal to you. The EKG shows no AV-block.

24. Would you stop or reduce the dosages of her metoprolol?

- yes (stop or reduce)
- no (keep the same dosage)

13. Case vignette 3

(same text) Another five years later, Mrs. A, now 78 years old, is moved to a nursing home. She has been

diagnosed with Alzheimer’s disease and is strongly dependent on support for her activities of daily living

such as dressing, personal hygiene and feeding. She only hardly walks on the rollator and has lost 5kg of

weight in the past six months. She still takes her aspirin (100mg/d), atorvastatin (40mg/d) and

metoprolol (100mg/d) regularly . Her left ventricular function is still normal with no signs of heart failure

or arrythmia. Her vital parameters (pulse and blood pressure) are normal to you. The EKG shows no AV-block.

25. Since you replied "yes" to the previous question, how would you change the dosage?

- reduce dose
- stop medication (= 100% dose reduction, no matter if tapering or direct stop)

14. Case vignette 3

(same text) Another five years later, Mrs. A, now 78 years old, is moved to a nursing home. She has been

diagnosed with Alzheimer’s disease and is strongly dependent on support for her activities of daily living

such as dressing, personal hygiene and feeding. She only hardly walks on the rollator and has lost 5kg of

weight in the past six months. She still takes her aspirin (100mg/d), atorvastatin (40mg/d) and

metoprolol (100mg/d) regularly . Her left ventricular function is still normal with no signs of heart failure

or arrythmia. Her vital parameters (pulse and blood pressure) are normal to you. The EKG shows no AV-block.

26. Would you stop or reduce the dosages of her metoprolol if she were complaining of dizziness when standing up?

- yes (stop or reduce)
- no (keep the same dosage)

15. Case vignette 3

(same text) Another five years later, Mrs. A, now 78 years old, is moved to a nursing home. She has been

diagnosed with Alzheimer’s disease and is strongly dependent on support for her activities of daily living

such as dressing, personal hygiene and feeding. She only hardly walks on the rollator and has lost 5kg of

weight in the past six months. She still takes her aspirin (100mg/d), atorvastatin (40mg/d) and

metoprolol (100mg/d) regularly . Her left ventricular function is still normal with no signs of heart failure

or arrythmia. Her vital parameters (pulse and blood pressure) are normal to you. The EKG shows no AV-block.

27. Since you replied "yes" to the previous question, how would you change the dosage if the patient were complaining of dizziness when standing up?

- reduce dose
- stop medication (= 100% dose reduction, no matter if tapering or direct stop)

28. What are your reasons for stopping or reducing the metoprolol in this situation? (multiple answers possible)

- her age
- frailty (in her case defined by the unintentional weight loss, low level of activity , slow gait speed)
- Alzheimer’s disease
- limited life expectancy
- lack of indication
- side-eﬀects (in her case dizziness when standing up)
- other (please specify)

16. Relevant factors for discontinuing or reducing beta blockers

Last, we would like to know which factors are important for you, when you are

deciding if you want to stop or reduce a beta blocker29. How important are the following factors when you consider stopping or reducing a beta blocker? (Very unimportant, unimportant, neutral, important, very important)

- Existence of guidelines to stop/reduce
- No clear indication
- Recommendations from involved specialists
- Benefit/harms of continuing
- Benefit/harms of stopping
- Previous experiences when stopping or reducing
- Patient’s preference
- Patient’s quality of life

Are there other factors that are important for you when you consider stopping or reducing a beta blocker?

(please specify)

**The male version of the vignettes was identical in all clinical details, with only the patient designation (name and honorific) changed from “Mrs A” to “Mr A”.**

# eAppendix 3: Reasons cited by GPs as important for deprescribing beta blockers

| **Factor** | **Female vignettes (n)** | **Male vignettes (n)** |
| --- | --- | --- |
| Patient’s quality of life | 98.7% (78) | 96.7% (88) |
| Benefits / harms of stopping | 97.4% (76) | 85.7% (78) |
| Benefits / harms of continuation | 92.4% (73) | 87.1% (81) |
| No clear indication | 84.8% (67) | 87.0% (80) |
| Specialists’ recommendation | 83.5% (66) | 84.9% (79) |
| Existence of guidelines | 78.5% (62) | 82.8% (77) |
| Patient’s preferences | 79.7% (63) | 75.6% (68) |
| Previous experience with stopping (GP level) | 65.4% (51) | 69.6% (64) |
